# SCAP Interacts with Trim27 to Promote Vascular Neointimal Hyperplasia by Regulating the Stability and Ubiquitination of IκBα

**DOI:** 10.14336/AD.2025.0584

**Published:** 2026-08-04

**Authors:** Yingcheng Yao, Xing Wang, Chengcheng Xuan, Da Li, Wenqin Huang, Li Wei, Xiong Z Ruan, Danyang Li, Yaxi Chen

**Affiliations:** ^1^Centre for Lipid Research & Chongqing Key Laboratory of Metabolism on Lipid and Glucose, Key Laboratory of Molecular Biology for Infectious Diseases (Ministry of Education), Institute for Viral Hepatitis, Department of Infectious Diseases, the Second Affiliated Hospital, Chongqing Medical University, Chongqing, China.; ^2^John Moorhead Research Laboratory, Centre for Nephrology, University College London Medical School, Royal Free Campus, University College London, London NW3 2PF, UK.

**Keywords:** SCAP, neointimal hyperplasia, vascular smooth muscle cell, Trim27, IκBα

## Abstract

Pathological vascular remodeling and intimal hyperplasia after vascular injury are representative pathological processes in age-associated vascular diseases. Previous data from our laboratory have indicated that sterol regulatory element-binding protein (SREBP) cleavage-activating protein (SCAP) contributes to physiological angiogenesis during embryonic development. However, the role of SCAP in neointima formation is not fully understood. Here, we aimed to explore the mechanisms of SCAP in the proliferation and migration of vascular smooth muscle cells (VSMCs) during neointima formation after injury. We utilized three types of transgenic (Tg) mice to demonstrate that SCAP participates in the regulation of injury-induced neointima formation in the vascular wall by promoting the proliferation and migration of VSMCs. This novel function of SCAP is associated with the activation of the NF-κB/MMP2/9 signaling pathway. Importantly, we reported for the first time that SCAP activates the NF-κB pathway by promoting Trim27-mediated ubiquitination of the IκBα protein and accelerating its degradation, consequently activating MMP2/9 transcription, which resulted in migration and proliferation of VSMCs. We thus propose that SCAP/IκBα/NF-κB axis is a novel signaling pathway involved in the regulation of neointimal hyperplasia, and targeting this axis may have implications for preventing neointimal hyperplasia-related diseases.

## INTRODUCTION

As the aging process proceeds, the significant alteration in vascular structure and function heightens the risk of vascular injury, consequently predisposing to intimal hyperplasia [1]. Neointimal hyperplasia is a common pathological characteristic in diverse vascular remodeling diseases, such as atherosclerosis, restenosis after angioplasty and stent placement, arterial occlusive disease, and transplant arteriosclerosis [2], and it is mainly defined as the abnormal migration and proliferation of vascular smooth cells (VSMCs) [3-6]. The causes of these diseases involve many intracellular signaling pathways and molecules [7]. Although the clinical significance of neointimal hyperplasia is globally recognized, its molecular and cellular mechanisms remain unclear.

SREBP cleavage-activating protein (SCAP), a membrane protein located in the endoplasmic reticulum (ER), is a sterol-sensing protein that chaperones SREBP-1 and SREBP-2 from the ER to the Golgi apparatus to maintain intracellular cholesterol homeostasis by regulating the transcription of 3-hydroxy-3-methylglutaryl CoA reductase (HMG-CoAR) and the low-density lipoprotein receptor (LDLR) [8, 9]. Our previous studies have shown that intervening in the expression of SCAP in VSMCs ameliorated the formation of atherosclerosis by inhibiting the foam cell formation derived from VSMCs. Moreover, we have observed that the number of VSMCs in the atherosclerotic plaque is related to SCAP expression [10, 11]. More importantly, VSMC-specific inactivation of SCAP in mice resulted in embryonic lethality due to impaired vascularization of the embryo proper and the labyrinthine layer in the placenta [6, 12]. These findings suggested that SCAP is implicated in the migration and proliferation of VSMCs. The physiological functions of SCAP have been extensively investigated, and this molecule plays a critical role in the development and cell proliferation of various organs. However, the role of SCAP in neointimal hyperplasia, which is a typical cell proliferative disease, has not been fully elucidated to date.

Cell proliferation and migration are critical in controlling the turnover of intracellular proteins. The ubiquitin-proteasome system (UPS) is at the core of protein turnover as the main effector of short- to medium-term degradation of proteins [13, 14]. Recently, several deubiquitinating enzymes and ubiquitin-conjugating enzymes, including USP10 and UbcH10, have been shown to be involved in the proliferation and migration of VSMCs [15]. Tripartite motif containing 27 (Trim27), a member of TRIM family, also known as Ret finger protein (RFP), was first identified as being fused to the Ret proto-oncogene. This protein possesses E3 ubiquitin ligase and SUMO E3 ligase activities and participates in cell proliferation, signaling pathway regulation and inflammatory responses by mediating the ubiquitination of IκBα and other proteins [16]. Trim27 and Trim28 have been reported to form a complex that jointly regulates the phenotypic switching of human VSMCs and the expression levels of platelet-derived growth factor receptor β (PDGFRβ) [17].

Herein, we found that the protein expression of SCAP was upregulated in VSMCs during neointimal hyperplasia after vascular injury in both mice and humans. We generated VSMC-specific (using the SM22α promoter) SCAP knockdown (KD), and overexpression (OE) mouse models and ligated their carotid arteries to establish a vascular injury model to investigate the underlying mechanism by which SCAP affects VSMC repair after flow-induced arterial injury both in vivo and in vitro.

## MATERIALS AND METHODS

### Human samples

The human samples were a gift from Professor Kong Wei, Department of Physiology and Pathophysiology, School of Basic Medical Sciences, Peking University. Three carotid artery specimens were obtained from hypertensive patients undergoing carotid endarterectomy. And three control left mammary arteries were derived from patient’s surgical donor arteries. The study was approved by the Ethical Committee and adhered to the Declaration of Helsinki. Informed consent was obtained from all patients.

### Animals

All mice were maintained on a C57BL/6J background. The D443N mutation in SCAP (located within its sterol-sensitive domain) results in sterol resistance due to a guanine-to-adenine substitution, which disrupts sterol-dependent regulatory mechanisms. This mutation stabilizes the SCAP-SREBP complex, promotes its translocation from the ER to the Golgi, and enhances proteolytic processing of SREBPs, even under sterol-replete conditions [18-20]. The SM22 promoter (pSM22) was amplified from murine genomic DNA via nested PCR, and the SCAP mutant amplified from the plasmid pTK-HSV-SCAP (D443N) was cloned into the pGL3-control vector to construct pGL3-SM22-SCAP (D443N). The SM22 promoter-driven SCAP overexpression mice (SCAP OE) were generated at the Nanjing Model Animal Center, China, through microinjection of prokaryotically expressed pGL3-SM22-SCAP [10]. Mice with conditional loxP-flanked SCAP (SCAPloxp/loxp) alleles and SM22α-Cre recombinase (Cre) mice (expressing Cre recombinase under the control of the mouse SM22α promoter) were purchased from The Jackson Laboratory (Bar Harbor, ME, USA). To generate mice with SM22α-Cre-specific SCAP knockdown (KD), SCAPloxp/loxp mice were crossed with SM22α-Cre mice to produce SM22α-Cre+ SCAPflox/+ offspring (referred to hereafter as SCAP KD mice), which were then intercrossed [11].

All mice used in this study were fed rodent chow under pathogen-free conditions. Each mouse was euthanized by intraperitoneal injection with an overdose of pentobarbital sodium (200 mg/kg) under deep anesthesia to obtain their samples. All animal experiments adhered to the National Institutes of Health Guide for the Care and Use of Laboratory Animals. These experiments were approved by the Institutional Animal Care and Use Committee of Chongqing Medical University (IACUC-CQMU-2023-0903).

### Carotid Artery Ligation and Endothelial Injury

Carotid artery ligation was performed as described previously [21]. In brief, 4-week mice were anesthetized with ketamine/xylazine (90 mg/kg and 10 mg/kg, respectively) via intraperitoneal injection. A 5-mm skin incision was made at the base of the neck and then blunt dissection until the left carotid artery was exposed. The left common carotid artery was carefully dissected from surrounding tissue and ligated with 6-0 silk (Ethicon K889H) sutures immediately proximal to the carotid bifurcation. The incisions were later closed, and the animals were monitored to ensure full recovery without neurological deficits. The contralateral common carotid artery served as an uninjured control. The mice were sacrificed at 6 weeks, and the carotid arteries were harvested for analysis.

### Cell culture

Mouse vascular smooth muscle cells (VSMCs) were obtained from American Type Culture Collection (ATCC, VA, USA). VSMCs were cultured in DMEM/F12 basal medium (Gibco, Thermo Fisher Scientific, Eugene, Oregon, USA) added with 10% fetal bovine serum (Natocor, Argentina) and 1% Penicillin streptomycin (Gibco, Thermo Fisher Scientific, Eugene, Oregon, USA). To establish VSMC SCAP overexpression cell lines, VSMCs were transfected with a lentivirus containing the full-length mouse SCAP cDNA (LV-SCAP) purchased from GeneCopoeia (Guangzhou, China) at a multiplicity of infection (MOI) of 50. A lentivirus expressing GFP (Lenti-NC) was used as a negative control. VSMCs were also transiently transfected with small interfering RNAs targeting SCAP (SCAPi), Trim27 (Trim27i) or a negative control siRNA, which sequences were shown on Supplementary Table S\1 in the Supplemental Material. The transfected cells were harvested for subsequent experiments. All cells were maintained at 37°C in a humidified atmosphere with 5% CO2.

### Immunofluorescence (IF) staining

Tissue sections or cells were fixed with 4% paraformaldehyde (PFA) for 15 minutes and then permeabilized with 0.1% Triton X-100 for 10 minutes. After that, nonspecific antigen was blocked with 3% bovine serum albumin (BSA) for 1 hour. The samples were then incubated with the following primary antibodies at 4°C overnight: anti-CD31 (CST, 3528, 1:400), anti-SCAP (Abcam, ab153933, 1:200), anti-OPN (R&D Systems, AF808, 1:200), anti-MMP2 (Santa, sc-13595, 1:100), anti-MMP9 (Santa, sc-21733, 1:100), anti-IκBα (Santa, sc-373893, 1:100), anti-p-IκBα (Santa, sc-8404, 1:100), anti-p-P65 (Proteintech, 82335-1-RR, 1:100), anti-Trim27 (Proteintech, 12205-1-AP, 1:200), anti-OPN (R&D Systems, AF808, 1:200), anti-Ubiquitin (Santa, sc-8017, 1:200), anti-rabbit IgG isotype control (Proteintech, 98136-1-RR, 1:200), anti-mouse IgG1 isotype control (Proteintech, 66360-1-Ig, 1:200). Fluorescence-labeled secondary antibodies in blocking buffer were applied for 2 hours at room temperature in the dark. Finally, the sections or cells were incubated with DAPI dye for 5 minutes, and images were captured using a Nikon laser scanning confocal microscope and analyzed with NIS-Elements AR Analysis software.

### Proximity ligation assay (PLA)

According to the manufacturer’s instructions (DUO 92008-30RXN, Sigma Aldrich) protein expression and localization of protein complexs were determined. Cell slides were fixed with 4% PFA (A500684, Sangon Biotech) for 15 minutes and permeabilized with 0.5% TritonX-100 (DH351-4, DINGGUO CHANGSHENG Biotechnology) for 15 minutes. Samples were blocked with Duolink blocking solution in an incubator at 37°C for 60 min, incubated with primary antibody solutions at 4°C for 8-12 hours, and the following day the incubation was continued at 37°C for 30 minutes. Next, the cells were treated with PLUS and MINUS PLA probe solutions for 60 minutes at 37°C, followed by incubation with ligase solutions for 30 minutes. The polymerase solution was added to the samples and incubated for 100 minutes. The slides were sealed with DAPI in situ fixative solution. Imaging was performed using a Nikon fluorescence microscope.

### Hematoxylin and eosin (H&E) staining

Paraffin-embedded sections (5 μm) were subjected to H&E staining. The slides were deparaffinized in xylene twice (5 minutes each), rehydrated using 100 %, 95 %, 80 %, and 75 % ethanol and distilled water (2 minutes each). The sections were then stained in hematoxylin (2 minutes; C0105; Beyotime, Shanghai, China), washed with tap water (10 minutes), counterstained in eosin (30 seconds; C0105; Beyotime, Shanghai, China), washed again with tap water (1 minute), dehydrated using 75 %, 80 %, 95 %, and 100 % ethanol (1 minute each), cleared with xylene twice (5 minutes each), and mounted with coverslips using neutral balsam(#G8590, Solarbio, Beijing, China). Finally, the tissues were scanned using a Pannoramic Flash DESK DX (3DHISTECH, Budapest, Hungary) and analyzed using Image J software.

### Immunohistochemical (IHC) staining

Paraffin-embedded sections (5μm) were subjected to dewaxing using xylene, rehydration using graded ethanol and microwave antigen retrieval using citric acid buffer (pH 6.0). Subsequent steps were carried out according to the manufacturer’s specifications for the IHC kit (PV-9001; ZSGB-BIO, Beijing, China). The sections were incubated with primary antibodies in blocking solution at 4°C overnight, including anti-OPN (R&D Systems, AF808, 1:400), and anti-PCNA (Proteintech, 81302-6-RR, 1:400). After that, the secondary antibodies in the IHC kit (ZSGB-BIO, Beijing, China) were used for 60 minutes at 37°C, followed by visualized using DAB solution (ZSGB-BIO, Beijing, China) and counterstained with hematoxylin for 8 minutes. Finally, all the sections were examined under a light microscope, and images were analyzed using Image Pro Plus software.

### Real-time quantitative PCR (RT–qPCR)

Total RNA was extracted using RNA isolate reagent (Vazyme, Nanjing, China), and then PrimeScript^TM^ RT Master Mix (Takara, Japan) was used to reverse transcribe total RNA to cDNA. Next, the cDNA products were performed to two-step PCR amplification using SYBR Green PCR Master Mix (Takara, Japan). The relative mRNA expression levels of target genes were calculated using 2^-ΔΔCt^ methodology. The housekeeping gene GAPDH was used as a reference gene. The primer sequences involved in this study were shown on Supplementary Table 2 in the Supplemental Material.

### Coimmunoprecipitation and Western blot

Protein A/G magnetic beads (B23202, Bimake) or agarose were incubated with IP antibodies overnight at 4°C. Platelet derived growth factor bb (PDGFBB)/SCAPi-treated VSMCs were lysed with Nonidet P40 buffer (BL653A, Biosharp), adding protease inhibitors and phosphatase inhibitors, and the protein supernatant was collected after centrifugation. Then, the supernatant was incubated with magnetic beads or agarose for 2 hours at 4°C to remove non-specific proteins. After that, the samples were incubated overnight with magnetic beads or agarose bound to the primary antibody. Finally, the magnetic beads or agarose were collected, washed with lysis buffer, and immunoprecipitates were eluted in 1×SDS sample buffer. Protein concentration was determined by the BCA kit (YAMEI, Shanghai, China). Precipitated proteins were mixed with protein loading buffer, boiled for 10 minutes at 100°C, and detected by western blotting.

Total cellular protein lysates were prepared in fresh RIPA buffer. Proteins were separated by SDS-PAGE (YAMEI, Shanghai, China) in a Bio-Rad Mini-protean apparatus (Bio-Rad, Hercules, CA, USA) and transferred to a PVDF membrane (Millipore, Billerica, MA, USA). The PVDF membranes were blocked with 3% BSA for 1 hour, and incubated at 4°C overnight with primary antibodies, including anti-SCAP (Abcam, ab153933, 1:3000), anti-MMP2 (Santa, sc-13595, 1:1000), anti-MMP9 (Santa, sc-21733, 1:1000), anti-IκBα (Santa, sc-373893, 1:1000), anti-p-IκBα (Santa, sc-8404, 1:1000), P65 (Proteintech, 8242, 1:2000), anti-p-P65 (Proteintech, 82335-1-RR, 1:2000), anti-Trim27 (Proteintech, 12205-1-AP, 1:2000), anti-Trim8 (Proteintech, anti-Trim8, 1:2000), anti-Trim22 (Proteintech, 13744-1-AP, 1:2000), anti-Trim24 (Proteintech, 14208-1-AP, 1:2000), anti-Ubiquitin (Santa, sc-8017, 1:1000), anti-IL1β (CST, 12242, 1:1000), anti-IL6 (Proteintech, 21865-1-AP, 1:2000), anti-TNFα (CST, 3707, 1:1000), and anti-GAPDH (Proteintech, 10494-1-AP, 1:2000). The following day, primary antibodies were detected using HRP-conjugated secondary antibodies: goat anti-rabbit (Proteintech, SA00001-2, 1:5000) and goat anti-mouse (Proteintech, SA00001-1, 1:5000). Finally, the membranes were visualized by using the High ECL Enhanced Western Blotting Substrate (YAMEI, Shanghai, China) and captured on an integrated chemiluminescence imaging system. GAPDH was used as a loading control.

### Immunoprecipitation-Mass Spectrometry (IP-MS)

Cells from the PDGFBB and CON groups were collected to prepare whole cell extracts for IP experiments using an SCAP antibody (Abcam, ab153933, 1:200), and then the magnetic beads were eluted to obtain the protein solution. Subsequently, proteins were separated using 7.5% SDS-PAGE, and the entire SDS-PAGE gel was cut after the protein samples ran 2cm out of the upper gel. The gel was stained with Coomassie Brilliant Blue staining solution, and the target lanes were excised for LC-MS/MS analysis. Mass spectral data were processed using SpectroMine software (version 4.2.230428.52329).

### EdU incorporation assay

To assess proliferation, VSMCs were evaluated using the BeyoClick™ EdU-555 Cell Proliferation Kit (Beyotime, Nanjing, China) according to the manufacturer's protocol. Briefly, 5×10^4^ VSMCs were seeded in 6-well plates and cultured overnight. After different treatments, the cells were incubated with DMEM/F12 medium containing EdU (20 μM) for 2 hours. The VSMCs were then fixed with 4% PFA for 15 minutes, permeabilized with 0.3% Triton X-100 for 10 minutes and incubated with a Click reaction cocktail (containing CuSO, Azide 555, reaction buffer, and additive) for 30 minutes in the dark. Next, the samples were washed and stained with Hoechst 33342 (1X) for 5 minutes and imaged using a Nikon laser scanning confocal microscope. The number of EdU-positive cells was quantified by Image J software.

**Figure 1.**
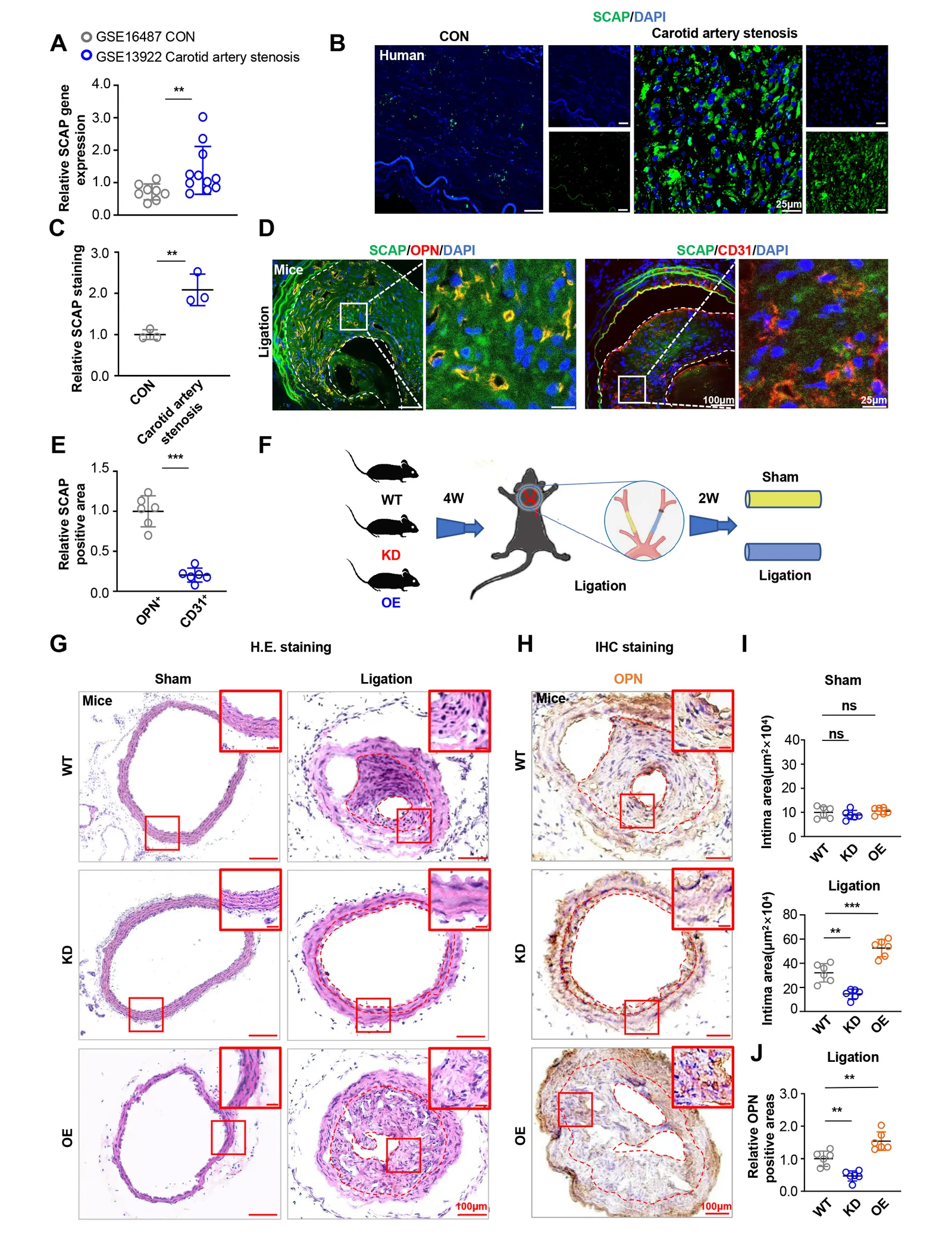
**SCAP in VSMCs may participate in neointima formation after injury**. (**A**) The expression of SCAP in neointimal tissue from patients with carotid artery stenosis and that in normal human left internal mammary artery. (**B**) Immunofluorescence staining of SCAP (green-FITC) in the neointima of patients with carotid artery stenosis and controls (n=3, Scale bar, 25μm). (**C**) Quantitative assessments of positive staining in (B). (**D**) Immunofluorescence staining of SCAP (green-FITC) and OPN or CD31 (red-TRITC) in the ligated sides of C57BL/6 mice (n=6, Scale bar, 100μm). Enlarged merge views are shown (Scale bar, 25μm). (**E**) Quantitative assessments of positive staining in (D). (**F**) SCAP KD and SCAP OE and their respective WT littermates were subjected to carotid artery ligation. (**G**) Hematoxylin and eosin staining of carotid artery cross-sections 2 weeks after injury (Scale bar, 100μm). Enlarged merge views are shown (n=6, Scale bar, 25μm). (**H**) Immunohistochemical staining of OPN in the ligated sides of mice (n=6, Scale bar, 100μm). (**I**) Statistical analysis of relative intimal areas. (**J**) Statistical analysis of relative OPN-positive areas. The number of each data represents biological replicates. Each data point, representing the number of biological replicates, is presented as mean ± SD. Groups were compared using Mann-Whitney U-test (A, C, E) or one-way ANOVA followed by Tukey’s test (I, J). *p < 0.05, **p < 0.01, ***p < 0.001, ns, no significant.

### Wound-healing assay

VSMCs treated with Trim27 small interfering RNA (Trim27i), PDGFBB, MG132 or negative control were seeded in 6-well plates. When the cells reached 90% confluence, they were scraped with a sterile 200 µL pipette tip. Subsequently, cell debris and floating cells were removed by washing twice with PBS, and the cells were maintained in serum-free medium. After 48 hours, the width of the wound was imaged using a microscope (Evos XL Core, Thermo Fisher Scientific). The wound areas were then quantified via Image J software.

### Transwell migration assay

This assay was detected in transwell inserts (Corning, USA) with 24-well cell culture plates composed of 8 μm pore polycarbonate filters. For the transwell migration assays, VSMCs treated with SCAPi, Trim27i, MG132, negative control and others were placed in the upper chamber with serum-free medium, while DMEM/F12 medium containing 10% FBS was added to the lower chambers. After incubation at 37 °C for 48 hours in a 5% CO_2_ incubator, the cells on the filter surface were fixed with 4% PFA and stained with crystal violet for 30 minutes. Finally, the cells in the upper chambers were removed using a cotton bud, and the cells on the undersides of the filters were counted under a light microscope (Evos XL Core, Thermo Fisher Scientific, Waltham, Massachusetts, USA) in five randomly selected fields per chamber.

### Statistics

Data are presented as mean ± SD. Statistical analyses used GraphPad Prism 8.0. Differences between two groups were compared using unpaired Student’s t-test (for normally distributed data, N ≥ 6) or Mann-Whitney U test (N < 6 or non-normal data). Comparisons among multiple groups used one-way ANOVA (normal data) followed by Tukey’s test. *P*<0.05 was considered statistically significant. *p < 0.05, **p < 0.01, ***p < 0.001; ns, no significant. Sample sizes were chosen based on preliminary data and were sufficient to detect significant effects. All outcome assessments and statistical analyses were performed by investigators blinded to group assignments.

## RESULTS

### SCAP in VSMCs may participate in neointima formation after injury.

To determine the role of SCAP in pathological vascular wall remodeling, we used the DESeq2 software and ComBat algorithms to normalize data from two GEO datasets (GSE16487 and GSE13922), ensuring their comparability. Specifically, we performed a comparison of the control groups from both datasets. For normalization, we calculated TPM (Transcripts per Kilobase of exon model per Million mapped reads) using the following formula, R_1_*10^9^/L_1_*R_total_. Then we found that the expression of SCAP was significantly upregulated in neointimal tissue from 11 patients with carotid artery stenosis compared to normal human left internal mammary artery (Fig. 1A). SCAP protein expression was also increased in the neointima of patients with carotid artery stenosis (Fig. 1B and 1C). To confirm these results, we generated a flow-induced arterial injury model by performing carotid artery ligation on male C57BL/6 mice (Supplementary Fig. 1A). Double immunofluorescence staining was used to assess SCAP expression in neointimal hyperplasia specimens. Among the cells involved in neointima formation, osteopontin (OPN) is a marker gene during the transition from contractile to synthetic phenotype of VSMCs, while vascular endothelial cells mainly express CD31. We found that SCAP was predominantly expressed in OPN-positive cells. In contrast, there was almost no abnormal expression in CD31-positive cells, indicating that SCAP overexpression was mainly localized in VSMCs in the intima of injured vessels, while the endothelial layer showed no significant expression compared to the neointima (Fig. 1D and 1E). To further investigate the underlying mechanism of SCAP in VSMC-related neointimal hyperplasia, we generated SCAP-knockdown (KD) and SCAP-overexpressing (OE) mice. The genotypes were confirmed by PCR, and vascular SCAP expression was validated (Supplementary Fig. 1B - D).

**Figure 2.**
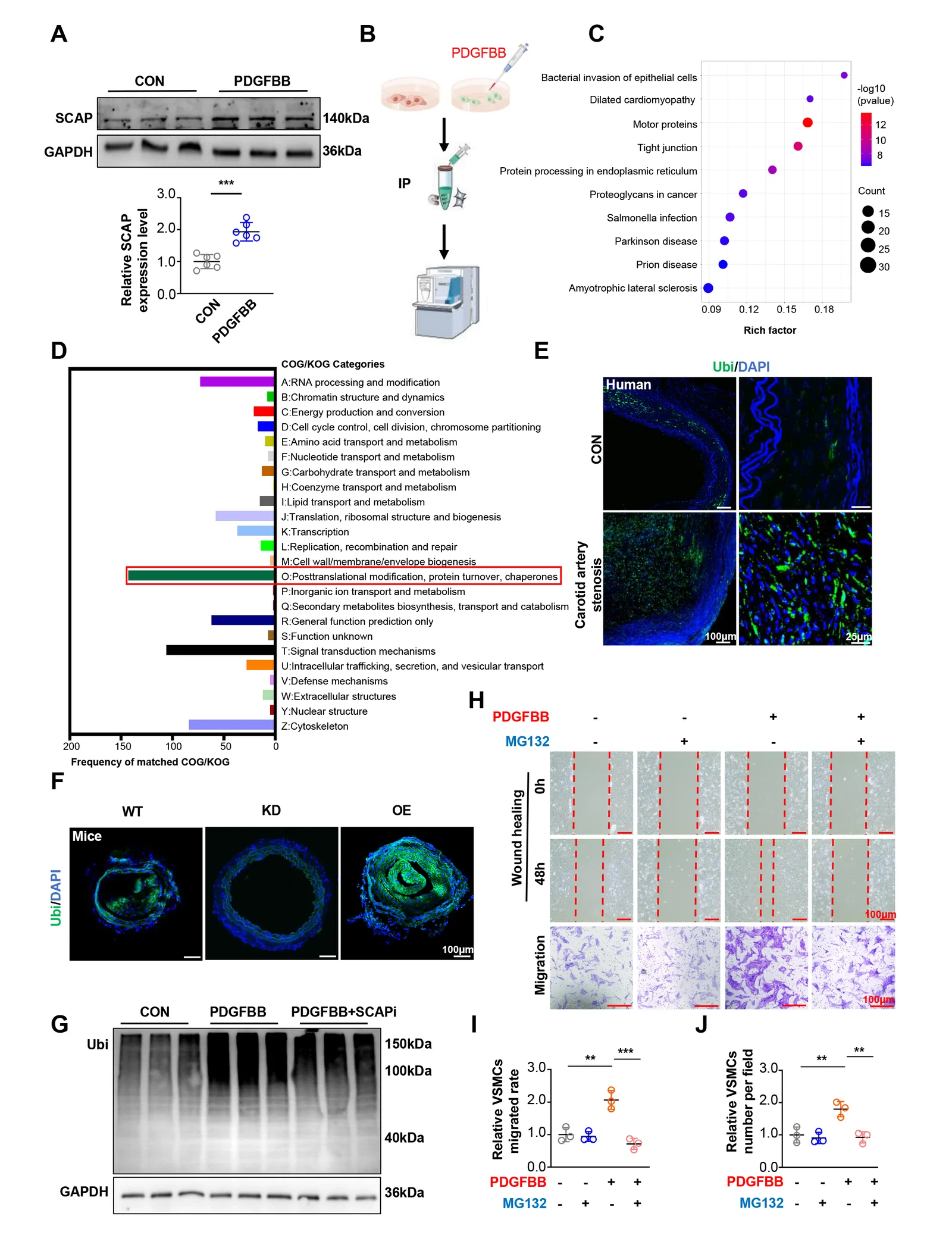
**SCAP influences the level of ubiquitinated proteins and proteasome activity through protein-protein interactions**. (**A**) VSMCs were cultured in medium containing 20 ng/ml PDGFBB for 24h, and SCAP protein expression was detected by Western blot (n=6). (**B**) Control and PDGFBB-treated VSMCs were immunoprecipitated with SCAP antibody, and the samples were processed for mass spectrometry analysis. (**C-D**) Kyoto Encyclopedia of Genes and Genomes (KEGG) and Clusters of Orthologous Groups (COGs) of the 273 proteins present in the PDGFBB-treated group but not in the control group. (**E**) Immunofluorescence staining of Ubi in clinical samples (n=3, Scale bar, 100μm). Enlarged merge views are shown (Scale bar, 25μm). (**F**) Immunofluorescence staining of Ubi in the ligated sides of mice (n=6, Scale bar, 100μm). (**G**) Western blot assessed the expression levels of Ubi in VSMCs treated with PDGFBB and SCAP siRNA (n=3). (**H**) Wound-healing and Transwell migration assay were used to estimate the migration ability of VSMCs under different treatments (n=3, Scale bar, 100μm). (**I-J**) Statistical analysis of the relative VSMCs migration rate (%) and the relative cell numbers per field (%) in (H). Each data point, representing the number of biological replicates, is presented as mean ± SD. Groups were compared using unpaired Student’s t-test (A) or one-way ANOVA followed by Tukey’s test (I, J). *p < 0.05, **p < 0.01, ***p < 0.001, ns, no significant.

Subsequently, carotid arteries were harvested from SCAP KD and OE mice 2 weeks post-ligation (Fig. 1F). H&E staining of the sham side showed no detectable intimal growth. After 2 weeks of ligation, a substantial increase in neointima was observed in SCAP OE mice, while SCAP KD mice exhibited markedly ameliorated neointima formation in the flow-induced arterial injury model (Fig. 1G and 1I). Immunohistochemical staining of OPN and proliferating cell nuclear antigen (PCNA) also confirmed that the thickness of the intima correlates with VSMC proliferation and migration, which may be related to SCAP expression level (Fig. 1H, 1J and Supplementary Fig. 1E). Due to the short modeling period of acute neointimal hyperplasia, we did not assess the impact of the mice's metabolic status on the local VSMC-driven pathological mechanisms. In vitro, we constructed an overexpression cell line using lentivirus infection in VSMCs. The efficiency was confirmed by Western blot analysis (Supplementary Fig. 1F). The proliferative index, measured by EdU staining, showed a notable positive correlation between fluorescence intensity and SCAP expression (Supplementary Fig. 1G). The migration evaluated with both scratch-wound and Boyden chamber chemotaxis assays showed that SCAP meditates cell proliferation and migration (Supplementary Fig. 1H through 1I). These data suggest that SCAP in VSMCs may play a crucial role in intima formation after injury.

### SCAP influences the level of ubiquitinated proteins and proteasome activity through protein-protein interactions.

Based on the results presented in this study and the previous knowledge of SCAP, we focused on its protein-protein interactions with other proteins. Western blot results demonstrated that SCAP protein expression was obviously increased in VSMCs after PDGFBB treatment (Fig. 2A). We immunoprecipitated SCAP and identified putative interacting proteins by mass spectrometry in the PDGFBB-treated group and the control group (Fig. 2B). Mass spectrometry analysis identified 273 proteins present in the PDGFBB-treated group but not in the control group. These proteins were searched against protein databases, including Clusters of Orthologous Groups (COGs) and Kyoto Encyclopedia of Genes and Genomes (KEGG), to identify their functional profiles (Fig. 2C and 2D). The major COG categories were post-translational modification, protein turnover, and chaperones (32.4%), followed by signal transduction mechanisms (Fig. 2D, total COG count: 26).

Since ubiquitination is closely associated with post-translational modification [22], we examined the overall ubiquitination levels in the neointima of patients with carotid artery stenosis and in mice with flow-induced arterial injury. Immunofluorescence staining using a polyubiquitinated substrate antibody revealed an increase in polyubiquitination levels in samples from the neointima of the patients with carotid artery stenosis (Fig. 2E and S2A). Interestingly, SCAP KD decreased the overall ubiquitination levels in neointimal lesions induced by carotid artery ligation, whereas SCAP OE increased these levels (Fig. 2F and Supplementary Fig. 2B). We evaluated the level of global ubiquitination in VSMCs, and, as shown in Figure 2G and S2C, VSMCs in the PDGFBB-treated group had a higher level of global ubiquitination than their counterparts, whereas a reduced level of ubiquitinated protein was apparent in VSMCs treated with SCAP siRNA (Supplementary Fig. 2D and 2E). This finding is consistent with the in vivo data presented above (Fig. 2F).

As an inhibitor of the proteasome, MG132 prevents the degradation of polyubiquitylated proteins [23]. Therefore, we utilized the proteasome inhibitor MG132 (MedChemExpress, HY-13259, 5μM for 48h) in our experiments and found that its use did not result in any additional differences in SCAP protein expression, regardless of whether VSMCs were treated with PDGFBB (Supplementary Fig. 2F). However, VSMC migration was promoted by PDGFBB treatment in wound-healing scratch experiments and in Transwell migration assays, and subsequent treatment with MG132 reversed this phenomenon (Fig. 2H through 2J). These results are largely consistent with the *in vivo* data. These observations indicate that SCAP affected VSMC migration through ubiquitination mechanisms, which in turn promotes neointima formation.

**Figure 3.**
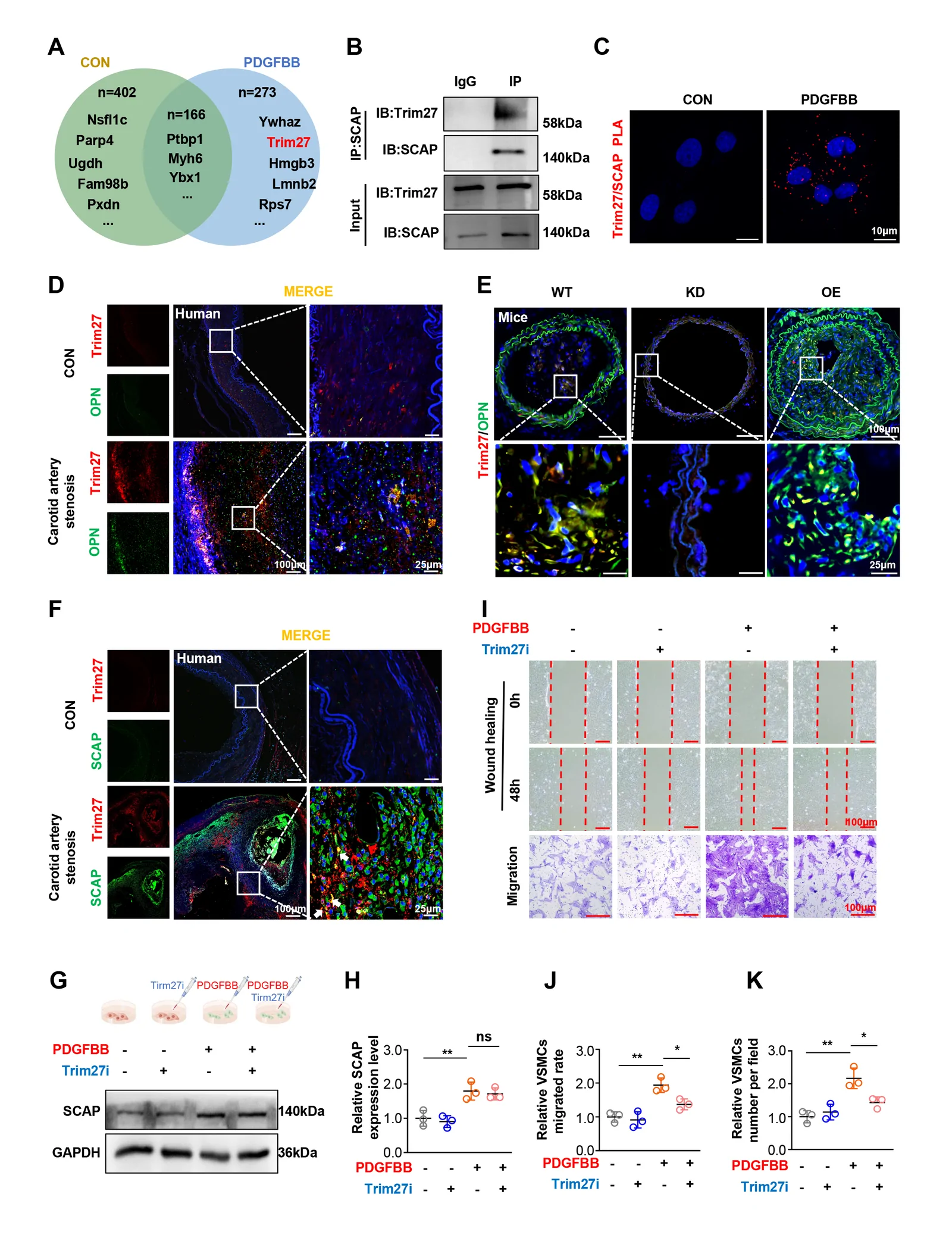
**SCAP regulates the process of ubiquitination through interactions with the Trim27 protein**. (**A**) Venn diagram of DEGs of VSMCs in control and PDGFBB-treated groups. (**B-C**) Interaction of SCAP and Trim27 in PDGFBB-treated VSMCs was analyzed by coimmunoprecipitation and proximity ligation assay (PLA) (n=3, Scale bar, 10μm). (**D**) The colocalization of Trim27 (red-TRITC) and OPN (green-FITC) in clinical samples (n=3, Scale bar, 100μm). Enlarged merge views are shown (Scale bar, 25μm). (**E**) The colocalization of Trim27 (red-TRITC) and OPN (green-FITC) in the ligated sides of mice by immunofluorescence staining (n=6, Scale bar, 100μm). Enlarged merge views are shown (Scale bar, 25μm). (**F**) The colocalization of Trim27 (red-TRITC) and SCAP (green-FITC) in clinical samples (n=3, Scale bar, 100μm). Enlarged merge views are shown (Scale bar, 25μm). (**G**) Western blot assessed the expression levels of SCAP in VSMCs treated with PDGFBB and Trim27 siRNA (n=3). (**H**)Relative quantitative statistics of protein expression in (G). (**I**) Wound-healing and Transwell migration assay were used to estimate the migration ability (n=3, Scale bar, 100μm). (**J-K**) Statistical analysis of the relative VSMCs migration rate (%) and the relative cell numbers per field (%) in (I). Each data point, representing the number of biological replicates, is presented as mean ± SD. Groups were compared using one-way ANOVA followed by Tukey’s test (H, J, K). *p < 0.05, **p < 0.01, ***p < 0.001, ns, no significant.

**Figure 4.**
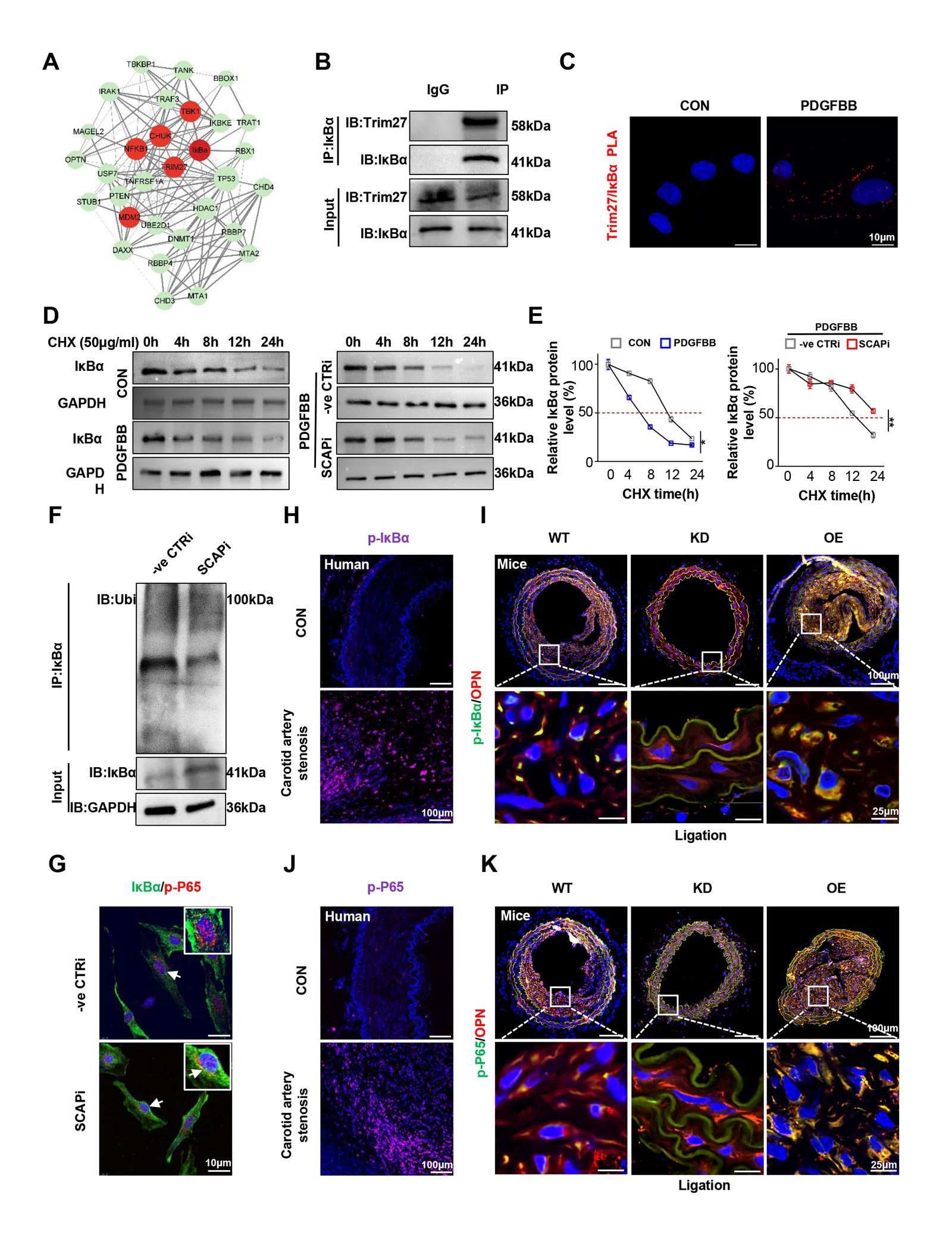
**SCAP affects the degradation of IκBα through the activation of Trim27**. (**A**) The protein-protein interactions (PPI) network analysis of Trim27 in STRING database. (**B-C**) Interaction of IκBα and Trim27 in PDGFBB-treated VSMCs was analyzed by coimmunoprecipitation and proximity ligation assay (PLA) (n=3, Scale bar, 10μm). (**D**) Western blot assessed the expression levels of IκBα in VSMCs treated with cycloheximide (CHX; 50 μg/mL) for 0, 4, 8, 12 and 24h (n=3). (**E**) Decay curve of IκBα was normalized to GAPDH and indicated the half-life of IκBα after SCAP expressional intervention. (**F**) Western blot of Co-IP performed with IκBα antibody to detect endogenous IκBα ubiquitination from indicated cells. (**G**) Identification of IκBα (green-FITC) and p-P65 (red-TRITC) in VSMCs of siSCAP treated group and -ve CTRi treated group by immunofluorescence to detect the combination between them (n=3, Scale bar, 10μm). (**H-I**) Immunofluorescence staining of p-IκBα in clinical samples (n=3, Scale bar, 100μm) and ligated sides of mice (n=6, Scale bar, 100μm). Enlarged merge views are shown (Scale bar, 25μm). (**J-K**) Immunofluorescence staining of p-P65 in clinical samples (n=3, Scale bar, 100μm) and ligated sides of mice (n=6, Scale bar, 100μm). Enlarged merge views are shown (Scale bar, 25μm). Each data point, representing the number of biological replicates, is presented as mean ± SD. Groups were compared using Mann-Whitney U-test (E). *p < 0.05, **p < 0.01, ***p < 0.001, ns, no significant.

### SCAP regulates the process of ubiquitination through interactions with the Trim27 protein.

We further analyzed our mass spectrometry data by Venn diagram analysis. The results showed that 273 proteins were uniquely present in the PDGFBB-treated group and absent in the control group. Among these proteins, Trim27 was the only protein closely associated with ubiquitination, and Trim27 may be essential for indirectly mediated ubiquitination by SCAP (Fig. 3A). We conducted coimmunoprecipitation (IP) experiments using equivalent amounts of SCAP immunoprecipitates to validate our mass spectrometry results (Fig. 3B). Further PLA analyses with VSMCs were consistent with the IP experiments, which suggested direct interaction between SCAP and the Trim27 proteins (Fig. 3C and S3A). Trim27 has been shown to influence migration and proliferation of VSMCs in vitro. Our immunofluorescence staining also demonstrated upregulation of Trim27 in proliferating VSMCs (OPN-positive) of carotid artery stenosis patients (Fig. 3D and S3B). Similar results were observed in the mouse arterial injury model (Fig. 3E and S3C). Importantly, the protein expression levels of Trim27 positively correlated with those of SCAP, and co-localization of SCAP with Trim27 was observed in the neointima of patients with carotid artery stenosis (Fig. 3F and Supplementary Fig. 3D). To exclude potential compensatory upregulation of related E3 ubiquitin ligases following changes in SCAP expression, we quantified protein levels of TRIM27, TRIM8, TRIM22, and TRIM24 in VSMCs subjected to SCAP knockdown. No statistically significant changes in the expression of these TRIM family members were detected, with the exception of TRIM27 (Supplementary Fig. 3E and 3F).

To determine whether Trim27 is a key molecule for SCAP-mediated downstream events, we silenced the expression of Trim27 using small interfering RNA (Trim27i) in VSMCs with or without PDGFBB treatment. The results suggested that PDGFBB treatment was instrumental in promoting Trim27 expression (Supplementary Fig. 3G and S3H), whereas RNAi suppressed its expression (Supplementary Fig. 3I and 3J). In parallel, Trim27i did not affect SCAP protein expression, regardless of PDGFBB treatment (Fig. 3G and 3H). Consistent with our earlier results (Fig. 2G), Trim27i could likewise reverse the decreased level of global ubiquitination induced by PDFGBB treatment (S3K and S3L). Moreover, the repression of cell migration was similarly demonstrated in VSMCs treated with Trim27i, as showen by wound-healing scratch experiments and Transwell migration assays (Fig. 3I through 3K), which is consistent with the earlier results under MG132 treatment (Fig. 2H and 2I). These results indicate that the interaction between SCAP and Trim27 is essential for the proliferation and migration of VSMCs during neointima formation.

### SCAP affects the degradation of IκBα through the activation of Trim27

Protein-protein interactions (PPIs) were predicted by using the online STRING database to identify the most suitable substrate for Trim27. On the basis of the mass spectrum data, we hypothesized that IκBα is likely the direct ubiquitination target of Trim27 during VSMC migration (Fig. 4A). The interaction between Trim27 and IκBα proteins was also validated by co-immunoprecipitation and PLA experiments (Fig. 4B and 4C), with a significant increase in the co-localization of Trim27 with IκBα observed after PDGF-BB treatment (Fig. 4C and Supplementary Fig. 4A).

Given that ubiquitination is a key mechanism regulating proteasome-mediated proteolysis of target proteins, we hypothesized that SCAP might affect the stability of IκBα. Cycloheximide (CHX) treatment was used to determine the half-life of IκBα, and the results confirmed our hypothesis that the turnover of IκBα was prolonged after SCAP siRNA transfection compared to the control groups, and the half-life of IκBα was decreased in cells treated with PDGFBB (Fig. 4D and 4E). The phosphorylation of IκBα leads to its ubiquitination and subsequent degradation by the proteasome. We then used Western blot analysis to examine the total protein and phosphorylated levels of IκBα. The phosphorylated level of IκBα was decreased in the SCAPi cell lines, reversing the increase in IκBα phosphorylation induced by PDGFBB treatment (Supplementary Fig. 4B and C). These data suggest that SCAP can affect IκBα protein stability in some way.

Immunoprecipitation of IκBα indicated that its ubiquitination level was decreased when SCAP was knocked down (Fig. 4F), indicating that the deletion of SCAP enhances IκBα protein stability through the loss of proteasomal-mediated protein degradation. IκBα phosphorylation is one of the major promoters of NF-κB liberation, and other reports have shown that the degradation of IκBα, especially through the ubiquitination (Ub)-mediated proteasome pathway, is required for NF-κB activation [24, 25]. Double immunofluorescence staining showed that SCAP knockdown markedly decreased the co-localization of p-P65 and IκBα, thus decreasing the nuclear conversion of p-P65 (Fig. 4G).

**Figure 5.**
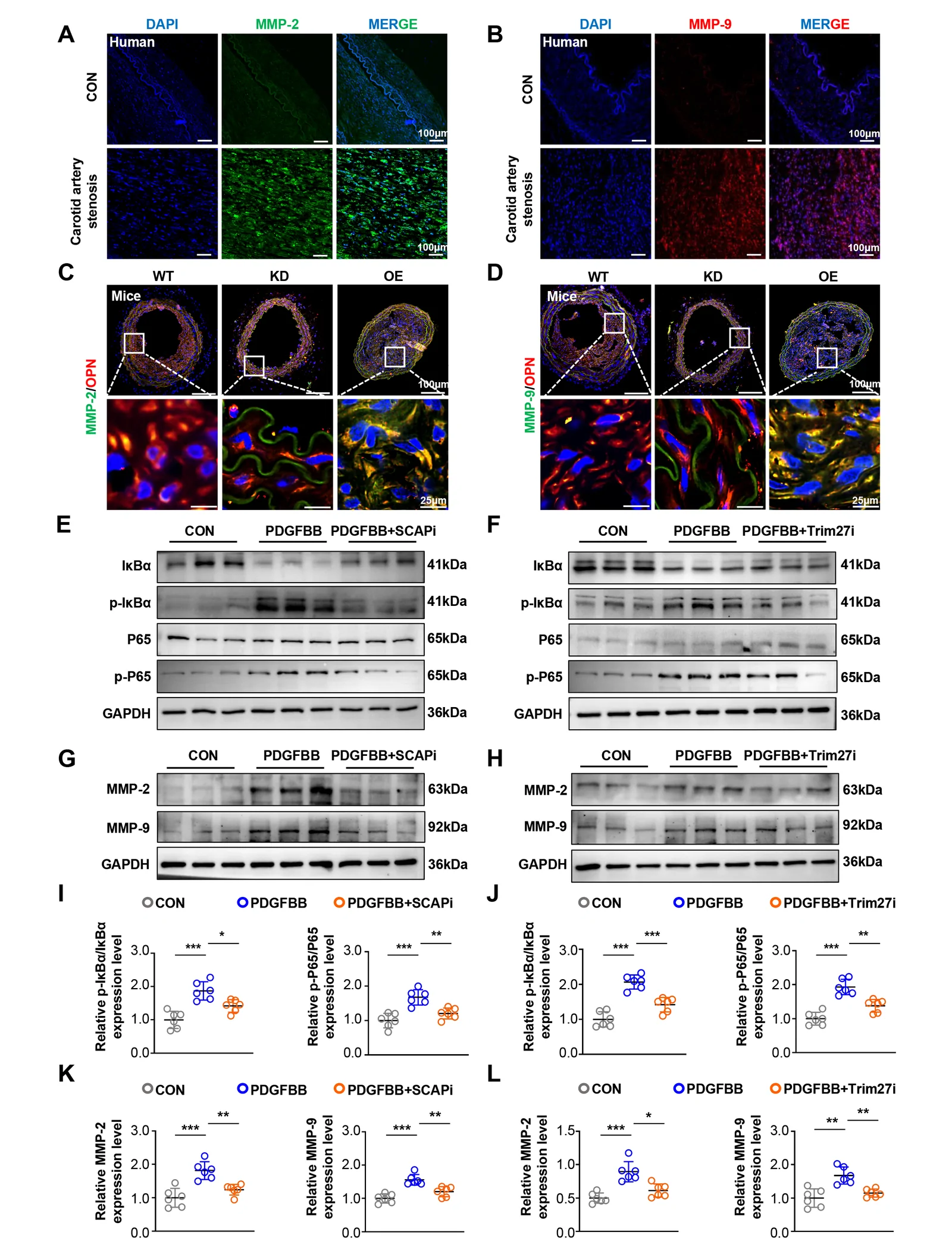
**SCAP affects cell proliferation and migration by affecting MMP2 and MMP9 expressions**. (**A-B**) Immunofluorescence staining of MMP-2 and MMP-9 in clinical samples (n=3, Scale bar, 100μm). (**C-D**) Immunofluorescence staining of MMP-2 and MMP-9 in ligated sides of mice (n=6, Scale bar, 100μm). Enlarged merge views are shown (Scale bar, 25μm). (E, G) Western blot assessed the expression levels of IκBα, p-IκBα, P65, p-P65, MMP-2 and MMP-9 in VSMCs treated with PDGFBB and SCAP siRNA (n=6). (F, H) Western blot assessed the expression levels of IκBα, p-IκBα, P65, p-P65, MMP-2 and MMP-9 in VSMCs treated with PDGFBB and Trim27 siRNA (n=6). (**I-L**) Relative quantitative statistics of protein expression in (E-H). Each data point, representing the number of biological replicates, is presented as mean ± SD. Groups were compared using one-way ANOVA followed by Tukey’s test (I-L). *p < 0.05, **p < 0.01, ***p < 0.001, ns, no significant.

We further verified this phenomenon in vivo. Immunofluorescence was used to analyze the expression levels and localization of p-IκBα in the neointima of patients with carotid artery stenosis or in mice with flow-induced arterial injury. The expression of p-IκBα was significantly upregulated in the neointima of patients with carotid artery stenosis (Fig. 4H and Supplementary Fig. 4F). In mice with flow-induced arterial injury, the expression of p-IκBα was related to SCAP expression levels (Fig. 4I and Supplementary Fig. 4G). Unsurprisingly, the expression of p-P65 was related to p-IκBα expression *in vivo* (Fig. 4J-K and Supplementary Fig. 4H-I). The same phenomenon was observed for SCAP RNAi-transfected VSMCs after PDGFBB treatment (Supplementary Fig. 4D - E).

Given the NF-κB signaling pathway plays an important role in the inflammatory response [26], we also evaluated the influences of SCAP or PDGFBB treatment on mRNA levels of inflammatory factors *in vitro* and *in vivo*. Unexpectedly, we did not observe any significant differences between the control group and the treated groups, either in cultured cells, vascular tissues, or within the systemic circulation of mice (Supplementary Fig. 4J-O). This finding indicates that SCAP does not significantly modulate the inflammatory response during neointima formation. While this result may initially appear paradoxical given NF-κB's canonical role, it is consistent with the highly complex and redundant nature of inflammatory signaling networks in VSMCs during intimal hyperplasia [27-29]. Multiple pathways beyond NF-κB, including recently identified mechanisms such as SOX10-driven macrophage-like VSMCs [30], contribute critically to cytokine production in intimal lesions. Consequently, isolated suppression of a single pathway like the IκBα/P65 complex—potentially regulated by SCAP via Trim27-mediated ubiquitination and degradation of IκBα—may yield limited cellular or systemic impact on the overall inflammatory cascade. We speculate that targeting SCAP may induce compensatory upregulation of alternative inflammatory signals. These data suggest that SCAP regulates the IκBα/P65 pathway by promoting the ubiquitination and degradation of the IκBα complex through upregulation of Trim27 expression, but it does not exert a measurable effect on the net inflammatory response in this context.

### SCAP affects cell proliferation and migration by affecting MMP2 and MMP9 expression

Previous studies showed that the phosphorylation and the translocation of P65 from the cytoplasm to the nucleus are key mediators in matrix metalloproteinase (MMP) activation [31, 32]; MMPs are extracellular matrix-related enzymes and important for regulating the function of VSMCs [33, 34]. We used immunofluorescence assays to examine the expression and distribution of MMP2 and MMP9 in specimens. The data showed that the expression of MMP2 and MMP9 was significantly upregulated in the neointima of patients with carotid artery stenosis (Fig. 5A-B and S5A-B), and their intensity was related to the protein level of SCAP (Fig. 5C-D and Supplementary Fig. 5C-D).

To verify the roles of SCAP and Trim27 in IκBα-induced upregulation of MMP proteins, we transfected VSMCs with SCAP RNAi or Trim27 RNAi. The Western blot results revealed that either SCAPi or Trim27i blocked the upregulation of MMP2 and MMP9 protein expression induced by PDGFBB treatment (Fig. 5E through 5L). This effect correlated with their ability to suppress PDGFBB-induced activation of the IκBα/P65 signal, which further supports our findings *in vivo*. Collectively, these data suggest that SCAP induced SMC proliferation and migration through activation of the IκBα/P65/MMP pathway.

### Disturbing the SCAP/Trim27/IκBα/MMP pathway partly rescues the phenotype caused by the abnormal expression of SCAP in VSMCs

**Figure 6.**
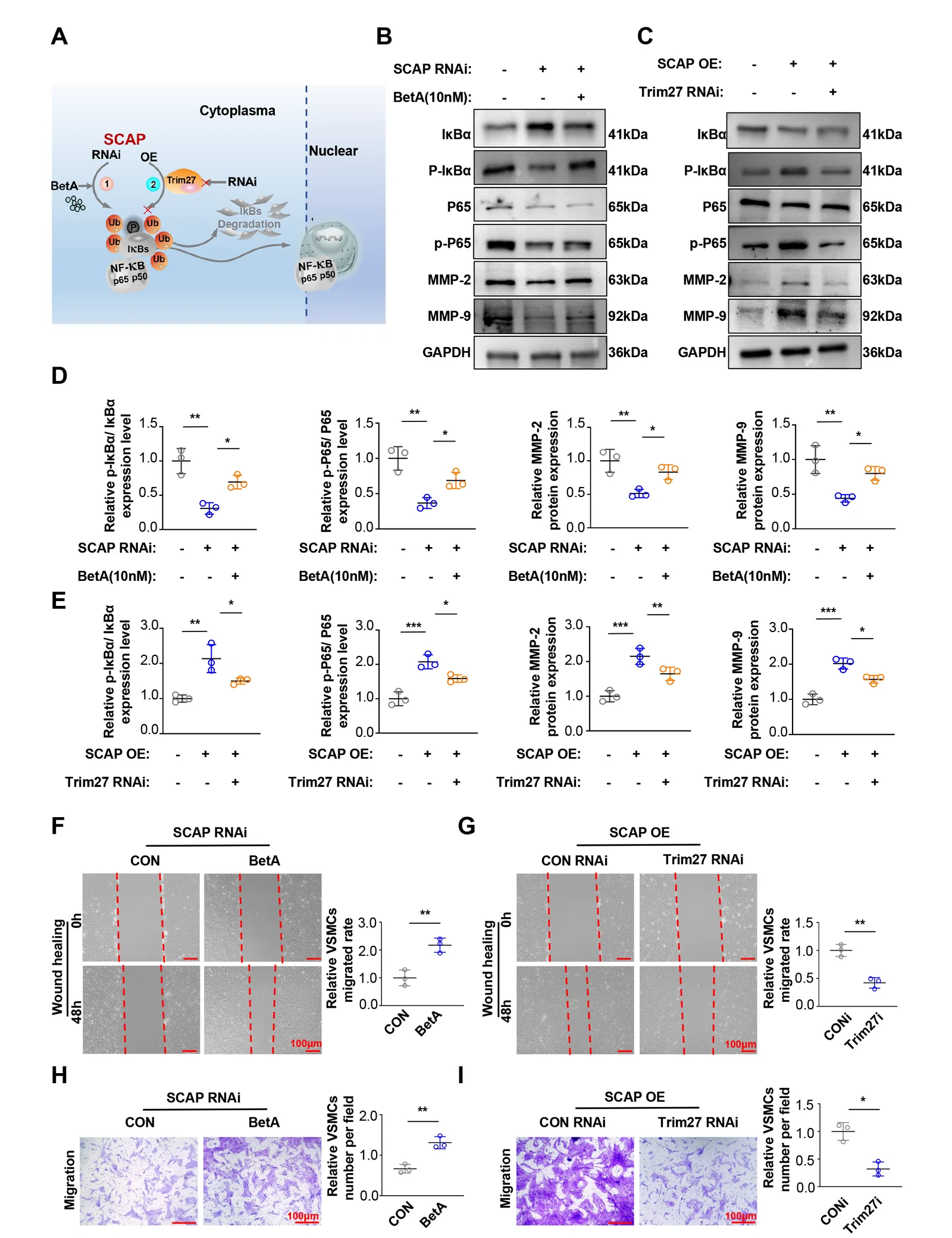
**Disturbing the SCAP/Trim27/IκBα/MMP pathway partly rescues the phenotype caused by the abnormal expression of SCAP in VSMCs**. (**A**) Treatments design to disturb the SCAP/Trim27/IκBα/MMP pathway. (**B**) Western blot assessed the expression levels of downstream in VSMCs treated with SCAP siRNA and BetA (n=3). (**C**) Western blot assessed the expression levels of downstream in VSMCs treated with siRNA Trim27 after overexpression of SCAP (n=3). (**D**) Relative quantitative statistics of protein expression in (B). (**E**) Relative quantitative statistics of protein expression in (C). (**F-I**) Wound-healing and Transwell migration assay showed the effects of rescue treatment on migration ability of VSMCs (n=3, Scale bar, 100μm). Each data point, representing the number of biological replicates, is presented as mean ± SD. Groups were compared using Mann-Whitney U-test (D-I). *p < 0.05, **p < 0.01, ***p < 0.001, ns, no significant.

Betulinic acid (BetA) is a novel activator of NF-κB that promotes the degradation of IκBα [35]. Trim27 RNAi was designed to disturb the ubiquitination of IκBα. BetA was used in siSCAP-treated VSMCs and Trim27 RNAi was used in the SCAP OE VSMC line (Fig. 6A). The IκBα/P65/MMP signaling pathway-related protein expression in BetA-treated or Trim27-silenced VSMCs was assessed by Western blot analysis. The results showed that treatment with BetA increased the phosphorylation of IκBα, promoted the activation of P65, and further ameliorated the inhibitory effect of siRNA transfection on MMP2/9. RNAi Trim27 treatment demonstrated that the activation of P65 mediated by overexpressed SCAP was neutralized by the reduced degradation of IκBα (Figs. 6B through 6E). Furthermore, the level of migration demonstrated by VSMC migration measured by wound-healing and Boyden chamber assays also showed correlations (Fig. 6F through 6I). To establish the causal role of MMP activity in SCAP-mediated migration, we performed functional rescue experiments using MMP inhibitors in SCAP-overexpressing VSMCs. Treatment with the broad-spectrum MMP inhibitor Doxycycline effectively reversed the SCAP overexpression-induced enhancement of VSMC migration and invasion (Supplementary Fig. 5E - G).

These results verified that SCAP affects the viability of Trim27 and further influences the nuclear transition of p-P65, promoting VSMC migration and proliferation with the expression of MMP2 and MMP9. Such functional specificity aligns with the pathological priority of VSMCs in vascular remodeling, where MMP-driven migration serves as the dominant mechanism underlying SCAP’s impact on intimal hyperplasia.

## DISCUSSION

We previously reported that the knockout of SCAP in VSMCs causes defects in vascular development [12, 13], suggesting a potential function of SCAP in physiological vascular remodeling. However, its role in pathological vascular remodeling has not been fully explored. In this study, we identified and validated that SCAP played a novel and key role in intimal hyperplasia. We found that PDGF or mechanical arterial injury can upregulate SCAP expression in VSMCs, along with the activation of the IκBα/P65/MMP2/9 pathways. Importantly, we demonstrated for the first time that the knockdown or overexpression of SCAP reduces or enhances VSMC proliferation and migration, as well as intimal hyperplasia, both *in vitro* and *in vivo.* Mechanistically, the effects of SCAP seem to be attributable to a Trim27-dependent mechanism. Our study provides strong evidence that IκBα is degraded by Trim27-mediated ubiquitination during neointima formation, and SCAP regulates IκBα protein stability by increasing Trim27 protein expression through protein-protein interactions. These results indicated that blocking SCAP or Trim27 regulates P65 activity and nuclear translocation to alleviate the process of neointima formation. While our study conclusively demonstrates a direct protein-protein interaction between SCAP and Trim27 and establishes that this interaction modulates Trim27's ubiquitination status, the precise molecular mechanism—specifically whether SCAP influences Trim27 at the transcriptional level, post-transcriptionally (e.g., mRNA stability/ translation), or primarily through post-translational stabilization—was beyond the scope of the current investigation.

Intimal hyperplasia, a representative complication of restenosis and atherosclerotic plaques, involves the thickening of the blood vessel wall in response to injury [36]. Notably, plasma cholesterol and inflammatory levels have been identified as key factors affecting restenosis and atherosclerotic plaques [37, 38]. We previously reported that cholesterol and inflammation resulted in abnormal intracellular activation of SCAP/HMG-CoA signaling pathway [39]. These data suggest that aberrant SCAP expression may be a potential mechanism linking metabolic disturbances to increased intimal hyperplasia. In this study, we further demonstrated that SCAP expression is linked with VSMC proliferation and migration. Our results are consistent with those reported by Dong et al. in *Esophageal Cancer* [40]. Specifically, VSMC-specific overexpression of SCAP promoted VSMC proliferation and migration, while inhibition or knockdown of SCAP in VSMCs led to a dramatic impairment of VSMC proliferation and migration and alleviation of intimal hyperplasia induced by injury or ligation, at least in part by mediating P65 activity and nuclear translocation. These results indicate that agents targeting SCAP, such as lycorine or betulin, may be new candidates for the clinical prevention of intimal hyperplasia [41, 42].

IκBα is an inhibitor of NF-κB, which is bound to nuclear factor κappa B (NF-κB) in the cytoplasm of unstimulated cells. The phosphorylation and degradation of IκBα trigger the nucleus translocation of NF-κB and the transcriptional expression of NF-κB-related genes. NF-κB is a family of transcription factors that includes RelA (P65), NF-κB1(P50), and NF-κB2(P52). Previous studies have demonstrated that NF-κB activation is enhanced in intimal lesions [43]. Pretreatment with the NF-κB inhibitor Bay 11-7082 counteracted VSMC phenotypic change, proliferation and migration in response to PDGFBB [44]. NF-κB has been shown to act as a critical regulator of inflammatory responses. However, based on our molecular analyses, SCAP does not seem to influence the inflammatory responses. In addition to inflammatory factors, MMP2/9 are other important target genes of NF-κB and are involved in cell proliferation and migration. With our study, we demonstrated that SCAP is involved in the regulation of MMP2/9 expression but not inflammatory factors. This finding could be due to the fact that VSMCs are not conventional inflammatory cells, the inherent limitations of in vitro experiments, or interactions between signaling pathways. This requires further exploration.

At present, it is widely accepted that one of the main pathogenic factors of restenosis is the inflammatory response triggered by the injured artery and the release of chemokines. However, there is still a lack of evidence to show the direct correlation between the formation of neointima and the degree of inflammation [45, 46]. Although it has been well established that inflammation is involved in the development of atherosclerosis, plaque formation, and instability, the specific role of inflammation in vascular occlusive diseases, such as neointima formation caused by mechanical damage to the artery after angioplasty or stenting, remains unclear [47]. This issue also explains why inhibition of SCAP does not reduce inflammation but can still improve neointima formation.

In human carotid complex atherosclerotic lesions, three genes, Trim22, Trim27, and Trim38, showed the highest expression among TRIM family genes. Studies have demonstrated that Trim27 forms a complex that jointly regulates the expression levels of contractile genes and PDGFRβ in human VSMCs [17]. Trim27 has been shown to destabilize the IκBα proteins through ubiquitination [48]. In contrast, the use of a Trim27 inhibitor has been found to reduce VSMC proliferation and migration, which further verified that IκBα protein stability is a promising therapeutic target for proliferative vascular diseases, especially those caused by hypercholesterolemia. Our studies have not yet clarified whether other E3 ubiquitin ligases could regulate VSMC proliferation and migration, which requires further investigation in the future.

Intimal formation appeared during normal development and aging as well as in the response of arteries to almost any imaginable injury, including atherosclerosis [4]. We emphasize that aging is an independent risk factor for coronary artery disease (CAD) and age-related vascular cell dysfunction, which drives pathological neointimal hyperplasia following vascular injury [49, 50]. The abnormal migration and proliferation of VSMCs—central to neointimal hyperplasia—are exacerbated in aging due to metabolic dysregulation [4, 51]. Hypercholesterolemia is closely associated with aging-related atherosclerosis and vascular remodeling [52]. Critically, aged cells exhibit profound cholesterol metabolic dysregulation [53], which may amplify the pathological activity of VSMCs. Our previous studies have demonstrated that genetic knockdown of SCAP in VSMCs attenuates atherosclerosis development [11], whereas SCAP overexpression exacerbates it [10]. Our study is the first to demonstrate that SCAP, a sterol-sensing protein central to cholesterol metabolism, directly regulates VSMC migration and proliferation, thereby modulating neointimal hyperplasia. These findings suggest that metabolic status—particularly sterol metabolic pathways governed by SCAP—should be considered a risk factor for intimal hyperplasia susceptibility in elderly patients.

In this study, we first demonstrated that SCAP acts as a driving factor in intimal hyperplasia, likely by regulating the activation of the IκBα/NF-κB pathways. In contractile VSMCs, SCAP expression is typically low. In contrast, in response to stimuli that promote VSMC dedifferentiation and proliferation, such as PDGFBB, SCAP expression is induced, thereby initiating the ubiquitination and degradation of IκBα and the subsequent enhanced nuclear translocation of P65, consequently activating MMP2/9 transcription, and resulting in migration and proliferation of VSMCs. To the best of our knowledge, we first identified SCAP as a novel and important factor in regulating intimal hyperplasia. The intervention of SCAP ameliorated VSMC proliferation and migration, shedding new light on a preventive strategy for neointimal hyperplasia and embryonic angiogenesis.

## Supplementary Materials

The Supplementary data can be found online at: www.aginganddisease.org/EN/10.14336/AD.2025.0584.


