# Supplementary material for: SCAP Interacts with Trim27 to Promote Vascular Neointimal Hyperplasia by Regulating the Stability and Ubiquitination of IκBα
Source: Aging Dis. 2026 Aug 4;17(5):2618–35. doi: 10.14336/AD.2025.0584 (PMC13437097; doi:10.14336/AD.2025.0584)
Supplement: Supplementary file 1 [file AD-17-5-2618-s.pdf]

SUPPLEMENTARY DATA

SCAP Interacts with Trim27 to Promote Vascular Neointimal Hyperplasia by Regulating the Stability and Ubiquitination of IκBα

Yingcheng Yao, Xing Wang, Chengcheng Xuan, Da Li, Wenqin Huang, Li Wei, Xiong Z Ruan, Danyang Li , Yaxi Chen

© 2025. Yao Y et al. Published online at http://www.aginganddisease.org/EN/10.14336/AD.2025.0584

SUPPLEMENTARY DATA

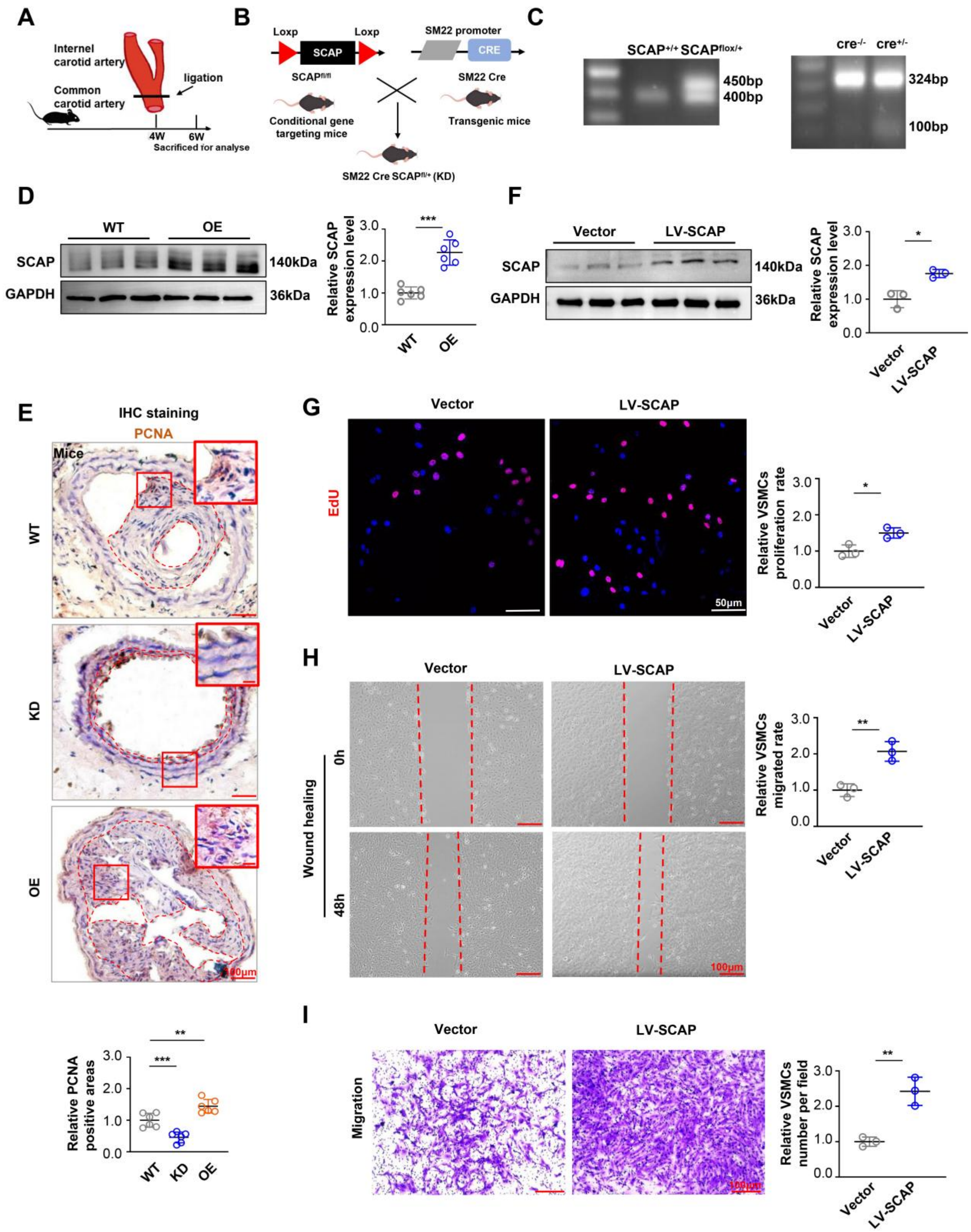

Supplementary Figure 1. Overexpression of SCAP promoted VSMC proliferation and migration in vitro and in vivo. *C57BL/6* mice were subjected to carotid artery ligation. (B) The SCAPloxp/loxp allele comprises loxP sites positioned

© 2025. Yao Y et al. Published online at http://www.aginganddisease.org/EN/10.14336/AD.2025.0584

flanking exon 1 of SCAP. Regarding the tissue-specific Cre transgenic mouse line, the expression of Cre recombinase is directed by the SM22 promoter. (C) Genotyping of tail tissue samples from offspring of SM22α-Cre+SCAPflox/+ mice via PCR revealed two genotypes: wild-type (WT) and knockout (KO). (D) Vascular SCAP protein expression in SCAP-overexpressing mice was assessed by Western blotting (n=6). (E)Immunohistochemical staining of PCNA in the ligated sides of mice (Scale bar, 100μm). Enlarged merge views are shown (n=6, Scale bar, 25μm). (F) Western blot assessed the expression levels of SCAP in lentivirus-treated VSMCs, verified the overexpression of SCAP in VSMCs (n=3). (G) Proliferation ability was analyzed by EdU staining (n=3, Scale bar,50μm). (H-I) Wound-healing and Transwell migration assay were used to estimate the migration ability (n=3, Scale bar, 100μm). Each data point, representing the number of biological replicates, is presented as mean ± SD. Groups were compared using Mann-Whitney U-test (F-I), unpaired Student's t-test (D), or one-way ANOVA followed by Tukey's test (E). *p < 0.05, **p < 0.01, ***p < 0.001, ns, no significant.

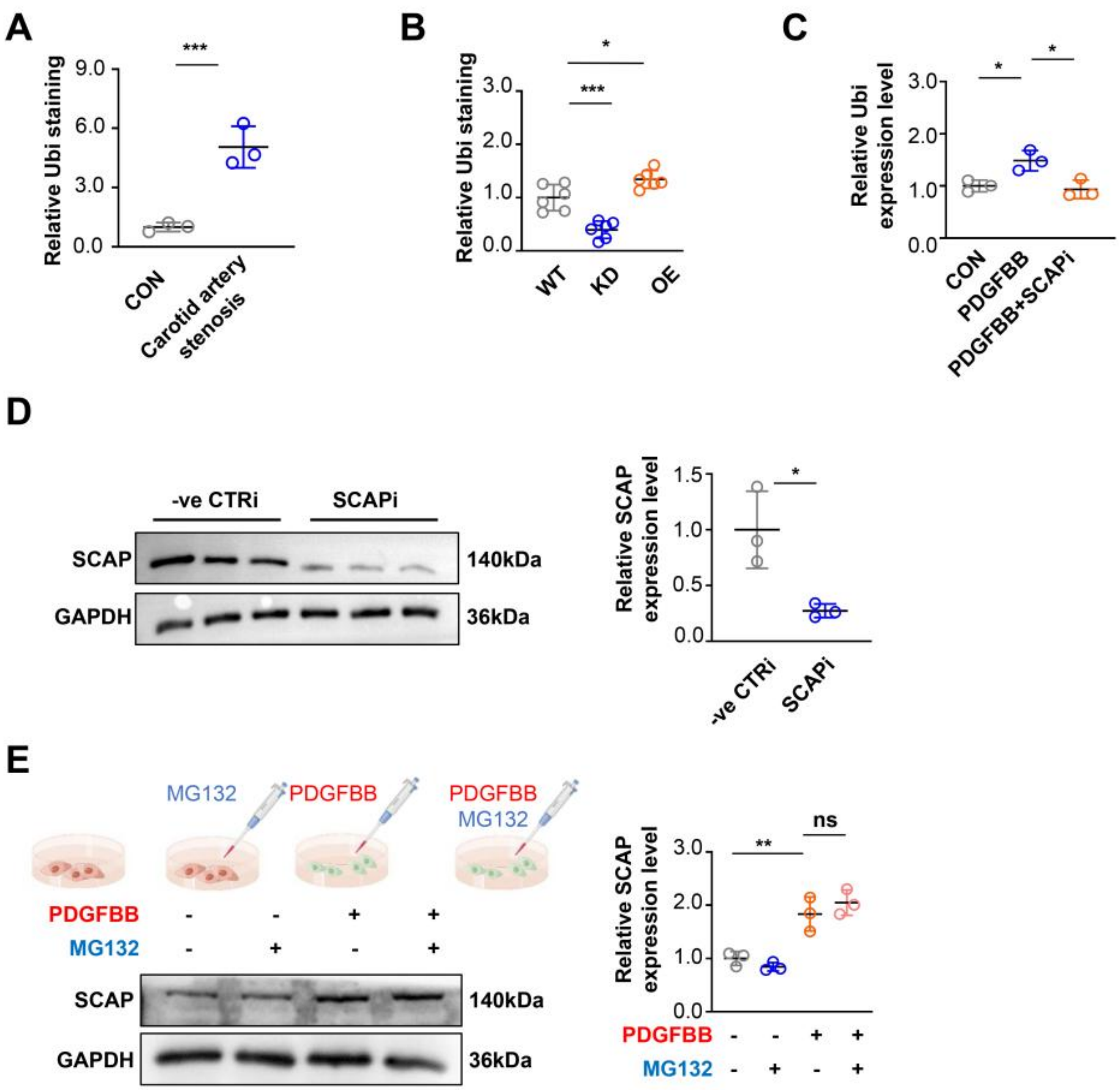

Supplementary Figure 2. SCAP is involved in the progression of ubiquitination. (A-C) Quantitative assessments of positive staining in Figure 2E-G. (D) Western blot assessed the expression levels of SCAP in siRNA-transfected VSMCs, verified the ablation of SCAP in VSMCs (n=3). (E) Western blot assessed the expression levels of SCAP in VSMCs treated with PDGFBB or MG132 (n=3). Each data point, representing the number of biological replicates, is presented as mean ± SD. Groups were compared using Mann-Whitney U-test (A, D) or one-way ANOVA followed by Tukey's test (B, C, E). *p < 0.05, **p < 0.01, ***p < 0.001, ns, no significant.

© 2025. Yao Y et al. Published online at http://www.aginganddisease.org/EN/10.14336/AD.2025.0584

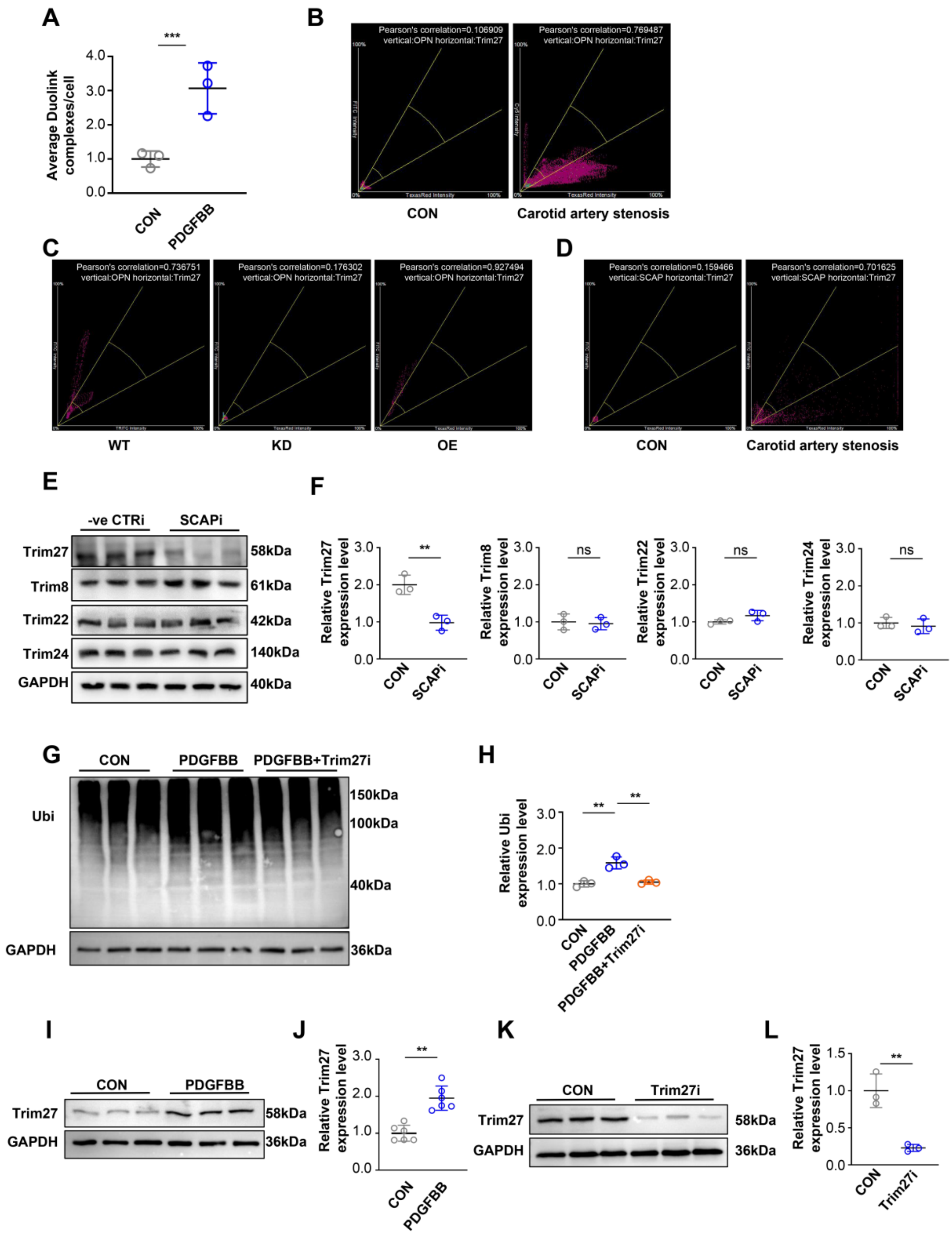

Supplementary Figure 3. SCAP regulates the ubiquitination process by interacting with Trim27. (A) Quantitative assessments of positive staining in Figure 3C. (B-D) Pearson's correlation statistical analysis of Figure 3D-F. (E) Western blot assessed the expression levels of Trim27, Trim8, Trim22, and Trim24 in VSMCs treated with *SCAP* siRNA (n=3). (F)

Relative quantitative statistics of protein expression of Ubi in (E). (G) Western blot assessed the expression levels of Ubi in VSMCs treated with PDGFBB and *Trim27* siRNA (n=3). (H) Relative quantitative statistics of protein expression of Ubi in (G). (I) Western blot assessed the expression levels of Trim27 in VSMCs treated with PDGFBB (n=6). (J) Relative quantitative statistics of protein expression of Trim27 in (I). (K) Western blot assessed the expression levels of Trim27 in siRNA-transfected VSMCs, verified the ablation of Trim27 in VSMCs (n=3). (L) Relative quantitative statistics of protein expression of Trim27 in (K). Each data point, representing the number of biological replicates, is presented as mean ± SD. Groups were compared using Mann-Whitney U-test (A, F, L), unpaired Student's t-test (J), or one-way ANOVA followed by Tukey's test (H). *p < 0.05, **p < 0.01, ***p < 0.001, ns, no significant.

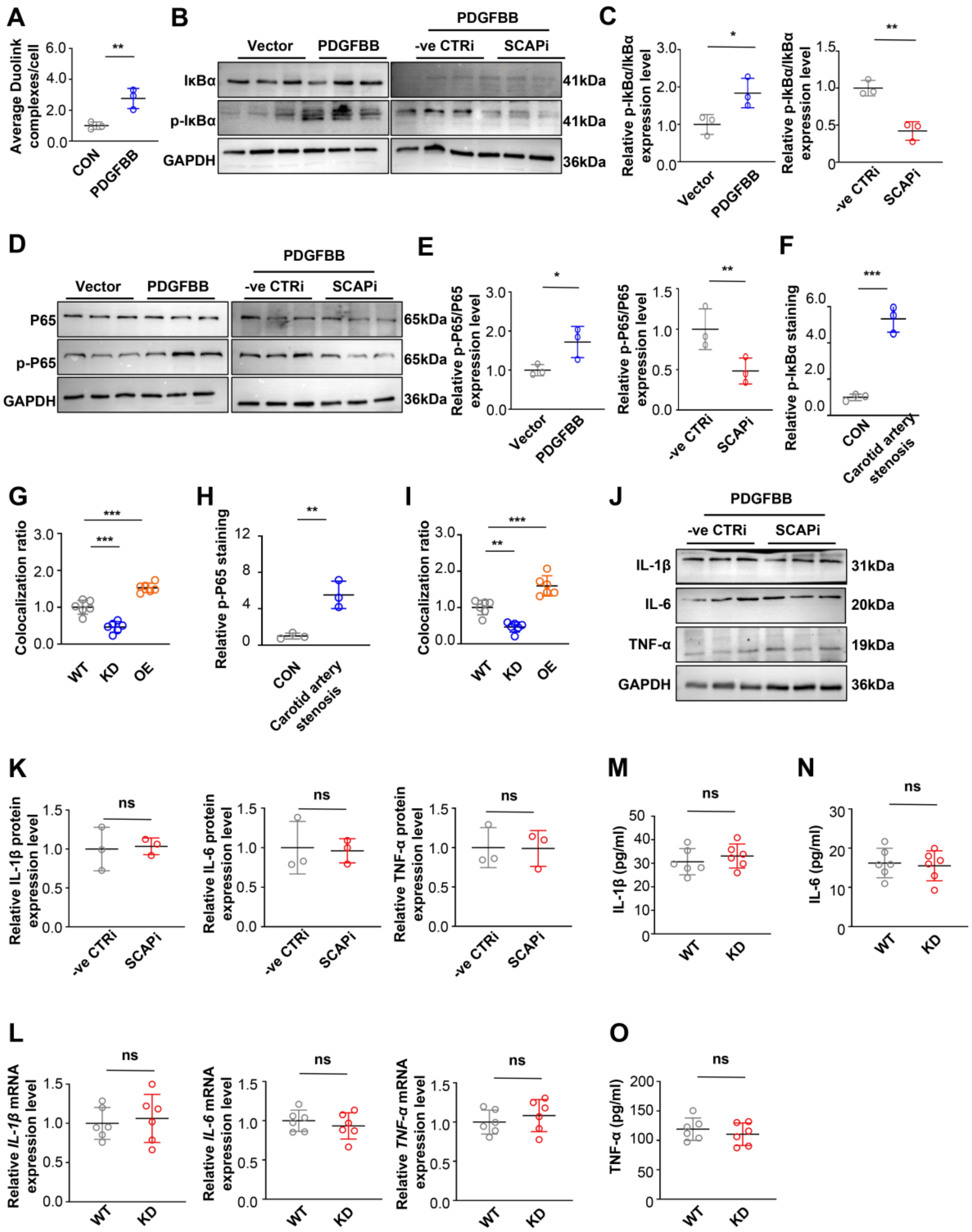

Supplementary Figure 4. SCAP is involved in VSMC proliferation and migration through the non-inflammatory effects of NF-κB. (A) Quantitative assessments of positive staining in Figure 4C. (B) Western blot assessed the expression levels of IκBα and p-IκBα in VSMCs treated with PDGFBB and *SCAP* siRNA (n=3). (C) Relative quantitative statistics of

© 2025. Yao Y et al. Published online at http://www.aginganddisease.org/EN/10.14336/AD.2025.0584

protein expression in (B). (D) Western blot assessed the expression levels of P65 and p-P65 in VSMCs treated with PDGFBB and *SCAP* siRNA (n=3). (E) Relative quantitative statistics of protein expression in (D). (F-I) Quantitative assessments of colocalization in Figure 4H-K. (J) Western blot of protein assessed the expression levels of IL-1β, IL-6 and TNF-α in VSMCs treated with PDGFBB and *SCAP* siRNA (n=3). (K) Relative quantitative statistics of protein expression of IL-1β, IL-6 and TNF-α in (J). (L) Relative quantitative statistics of mRNA expression of *IL-1β*, *IL-6* and *TNFA*. (M-O) Serum levels of IL-1β, IL-6 and TNF-α in ligated mice with different genotypes. Each data point, representing the number of biological replicates, is presented as mean ± SD. Groups were compared using Mann-Whitney U-test (A, C, E, F, H, K), unpaired Student's t-test (L, M, N, O), or one-way ANOVA followed by Tukey's test (G, I). *p < 0.05, **p < 0.01, ***p < 0.001, ns, no significant.

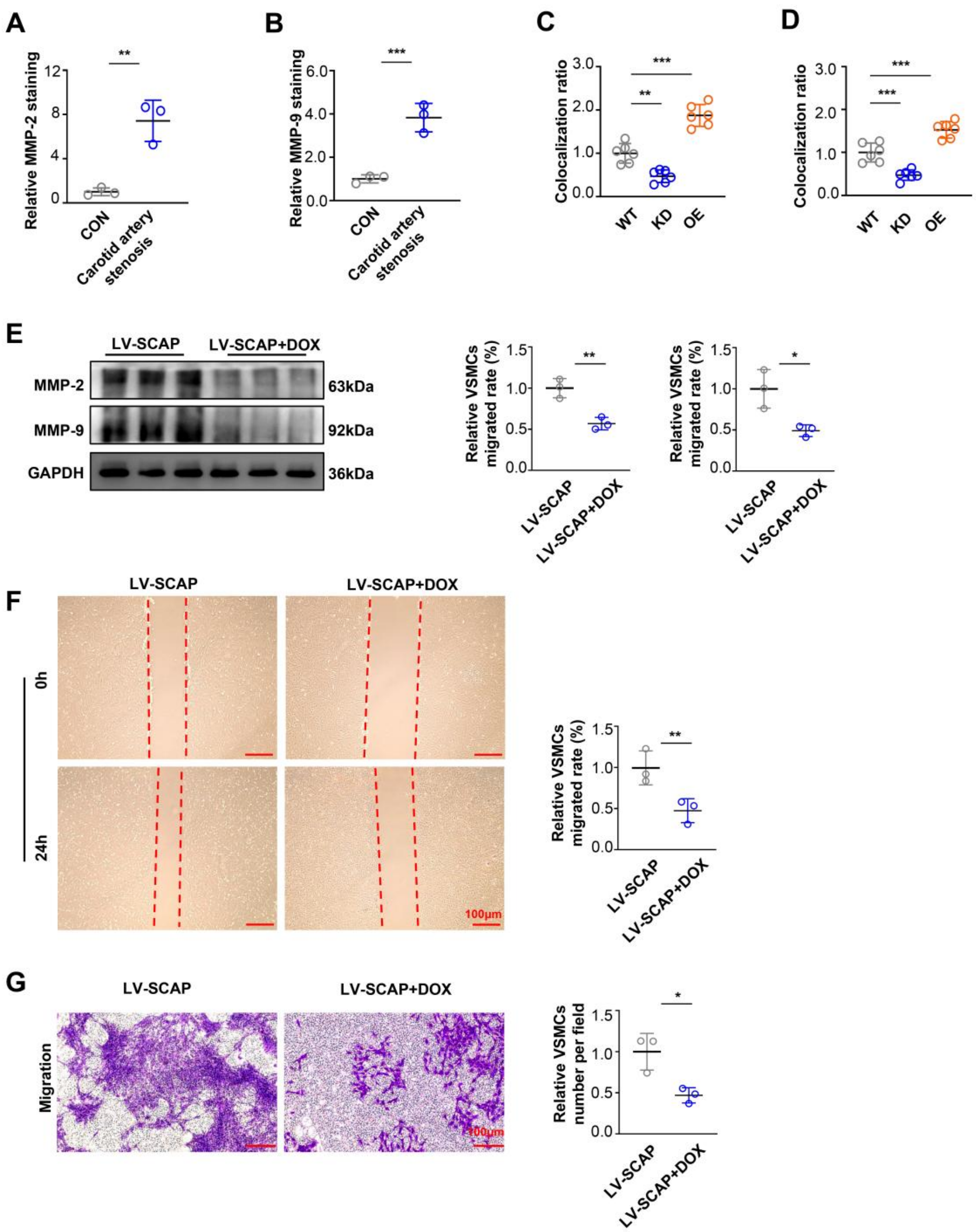

Supplementary Figure 5. MMP Inhibitors reverse SCAP-induced VSMC migration and proliferation. (A-D) Quantitative assessments of colocalization in Figure 5A-D. Quantitative assessments of positive staining in Figure 4C. (E) Western blot assessed the expression levels of MMP-2 and MMP-9 in in SCAP-overexpressing VSMCs treated with DOX (n=3). (F-G) Wound-healing and Transwell migration assay showed the effects of DOX on migration ability of SCAP-overexpressing VSMCs (n=3, Scale bar, 100μm). Each data point, representing the number of biological replicates, is

presented as mean ± SD. Groups were compared using Mann-Whitney U-test (A, B, E, F, G), unpaired Student's t-test (D), or one-way ANOVA followed by Tukey's test (C, D). *$p < 0.05$, **$p < 0.01$, ***$p < 0.001$, ns, no significant.

Supplementary Table 1. The siRNA sequences.

Target gene		Sequence (5' to 3')
Scap (Mouse)	sense	CCAUGGCGACAUUACCUUGUATT
	antisense	UACAAGGUAAUGUCGCCAUGGTT
Trim27 (Mouse)	sense	GAGUGAAAGACUUGAAGAATT
	antisense	UUCUUCAAGUCUUUCACUCTT

Supplementary Table 2. The primers used in RT-PCR.

Genes	Forward Primer Sequences (5'-3')	Reverse Primer Sequences (5'-3')
Il-1β	GCAACTGTTCCTGAACTCAACT	ATCTTTTGGGGTCCGTCAACT
Il-6	ATCTTTTGGGGTCCGTCAACT	TTGGTCCTTAGCCACTCCTTC
Tnf-α	CCCTCACACTCAGATCATCTTC T	GCTACGACGTGGGCTACAG
Gapdh	AGGTCGGTGTGAACGGATTTG	AGGTCGGTGTGAACGGATTTG

© 2025. Yao Y et al. Published online at http://www.aginganddisease.org/EN/10.14336/AD.2025.0584
